# Cost-effectiveness of drug consumption rooms in France: a modelling study

**DOI:** 10.1186/s12889-024-18909-9

**Published:** 2024-05-28

**Authors:** Anthony Cousien, Cécile Donadille, Laélia Briand Madrid, Gwenaëlle Maradan, Marie Jauffret-Roustide, Laurence Lalanne, Marc Auriacombe, Perrine Roux, Sylvie Boyer

**Affiliations:** 1 Université Paris Cité and Université Sorbonne Paris Nord, Inserm, IAME, F-75018 Paris, France; 2 Aix Marseille Univ, INSERM, IRD, SESSTIM, Sciences Economiques & Sociales de la Santé & Traitement de l’Information Médicale, ISSPAM, Marseille, France; 3 ORS PACA, Observatoire régional de la santé Provence-Alpes-Côte d’Azur, Marseille, France; 4 Centre d’Etudes des Mouvements Sociaux, Inserm U1276, CNRS UMR 8044, EHESS, Paris, France; 5 British Columbia Center on Substance Use (BCCSU), Vancouver, Canada; 6 Baldy Center on Law and Social Policy, Buffalo University, New York, USA; 7 Institut universitaire sur les dépendances, Université de Sherbrooke, Montréal, Québec Canada; 8 UMR 1329, Team Addictions, Centre de recherche en biomédecine de Strasbourg, Department of addiction medicine, University hospital of Strasbourg, Strasbourg, France; 9 UMR 6033, Bordeaux University, SANPSY, CNRS, Bordeaux, France

**Keywords:** Injecting drug users, Harm reduction, Cost-effectiveness, Modelling study, Supervised injection facility

## Abstract

**Background:**

People who inject drugs (PWID) experience many health problems which result in a heavy economic and public health burden. To tackle this issue, France opened two drug consumption rooms (DCRs) in Paris and Strasbourg in 2016. This study assessed their long-term health benefits, costs and cost-effectiveness.

**Methods:**

We developed a model to simulate two fictive cohorts for each city (*n*=2,997 in Paris and *n*=2,971 in Strasbourg) i) PWID attending a DCR over the period 2016-2026, ii) PWID attending no DCR. The model accounted for HIV and HCV infections, skin abscesses and related infective endocarditis, drug overdoses and emergency department visits. We estimated the number of health events and associated costs over 2016-2026, the lifetime number of quality-adjusted life-years (QALYs) and costs, and the incremental cost-effectiveness ratio (ICER).

**Results:**

The numbers of abscesses and associated infective endocarditis, drug overdoses, and emergency department visits decreased significantly in PWID attending DCRs (-77%, -69%, and -65%, respectively) but the impact on HIV and HCV infections was modest (-11% and -6%, respectively). This resulted in savings of €6.6 (Paris) and €5.8 (Strasbourg) millions of medical costs. The ICER of DRCs was €30,600/QALY (Paris) and €9,200/QALY (Strasbourg). In scenario analysis where drug consumption spaces are implemented inside existing harm reduction structures, these ICERs decreased to €21,400/QALY and €2,500/QALY, respectively.

**Conclusions:**

Our findings show that DCRs are highly effective and efficient to prevent harms in PWID in France, and advocate extending this intervention to other cities by adding drug consumption spaces inside existing harm reduction centers.

**Supplementary Information:**

The online version contains supplementary material available at 10.1186/s12889-024-18909-9.

## Background

People who inject drugs (PWID) experience many health problems. The burden of chronic viral infections is high in this population, with an estimated global prevalence of 17.8 and 52.3% for human immunodeficiency virus (HIV) and hepatitis C virus (HCV), respectively [1]. Bacterial infections also represent a major issue in PWID, with 6 to 32% reporting skin and soft tissue infections in the previous month [2]. Finally, 41.5% of PWID report overdosing during their lifetime [3]. All these health events have a huge impact on PWID life expectancy [4, 5] and quality of life [6].

Harm reduction measures have traditionally relied on access to sterile injection equipment through needle/syringe exchange programs, and opiate agonist therapy [7]. Some countries, such as Australia, Switzerland and Canada, have also implemented drug consumption rooms (DCRs). These are places where PWID can consume drugs in good hygienic and sanitary conditions, and access care adapted to their needs. Studies from Australia and Canada show that DCR bring many benefits, including a decrease in the risk of overdose and syringe sharing, improved access to care, and a positive impact on local drug-related violence and trafficking [8, 9].

In France, psychosocial and health services for PWID are usually delivered in harm reduction centers for people who use drugs, namely CAARUD. These services are financed by the national health insurance system (NHI) and are managed either by non-profit organizations or by public hospitals. Two DCRs were opened on an experimental basis in Paris and Strasbourg in 2016. The COSINUS cohort [10] was established the same year to assess the two DCRs’ effectiveness in PWID, especially in terms of fewer infection risk practices and adverse health events. Cohort participants were recruited between June 2016 and October 2018 in both DCRs and in CAARUDs in Paris, Strasbourg, Bordeaux and Marseilles and followed up to 12 months. Results showed that attending DCRs significantly reduced the probability of reporting injecting equipment sharing, abscesses, overdoses, and emergency department (ED) visits [11]. A sociological survey embedded in the evaluation also showed an improvement in regards to public safety [12] and social acceptance, especially among harm reduction and addiction professionals [13]. Furthermore, existing economic evaluation studies suggested that the intervention is cost-effective and in some settings even cost-saving (i.e., avoided medical costs because of avoided health events exceed the cost of DCR). However, all these studies have been conducted in North America settings (Canada and the United States) where the organization of the health system and the epidemiological situation are very specific [1, 14] and all used DCR effectiveness data from one single experience in Vancouver to simulate the impact of this intervention on long-term PWID health.

In the perspective of the potential extension of the DCR experiment to other cities in France, further information on their costs and efficiency is needed for decision-making by national health authorities.

Using a modelling approach combining data from the COSINUS cohort and the literature, this study aims to assess the long-term health benefits, costs avoided and cost-effectiveness of DCR compared to standard services offered in CAARUD in the France setting.

## Methods

### Analytic overview

We designed a stochastic, individual-based, continuous-time model to simulate health outcomes and related costs in hypothetical populations of PWID attending either the Paris or Strasbourg DCR over a 10-year period. The comparator harm reduction strategy was the situation where only CAARUD are present (i.e., the standard situation without DCR).

The model simulated the occurrence of the following health events: new HIV and HCV infections, abscesses and associated infective endocarditis (IE), overdoses, and ED visits. We stratified analyses by city (Paris and Strasbourg) to account for local specificity (e.g. characteristics of PWID and cost of DCR). We first estimated health outcomes and associated medical costs for both strategies over a 10-year horizon and then conducted the complete cost-effectiveness analysis over a lifetime horizon. In line with recommendations from the French National Authority for Health (HAS) [15], the cost-effectiveness analysis was conducted using the point of view of the national health system (irrespective of the financing body i.e. the Ministry of health or the NHI) and included both medical costs incurred by the NHI and the costs of establishing and running the two DCR, funded by public resources.

### Simulated populations

For both Paris and Strasbourg, we simulated two hypothetical populations: i) PWID attending the DCR (plus possibly CARRUD) over a 10-year period starting from the DCR opening date and ii) PWID attending no DCR (but possibly CARRUD) over the same period (See Additional file S1 for further details).

For both strategies, the number of new entrants in the model per unit of time, and their age and gender distribution were defined using DCR activity registers over the period 2016-2019 (Table 1).

**Table 1:** Key parameters of the model. A complete list can be found in Additional file S3

**Parameter**	**Value**	**Reference**
Rate of new entries in the attendee population in the Paris DCR		Based on DCR attendance data available for the period 2016-2019. We assumed a constant entry rate from 2019 onwards in the main analysis.
* 2016 (October to December)*	484	
* 2017*	402	
* 2018*	235	
* 2019 and beyond*	242	
Rate of new entries in the attendee population in the Strasbourg DCR		
* 2016 (November to December)*	153	
* 2017*	257	
* 2018*	227	
* 2019 and beyond*	298	
Dynamics of attendance in both DCRs (scenario S1)		COSINUS data, based on transitions observed between M0 and M6, and between M6 and M12.
* DCR attendance → Exit*	35.4%/year	
* Exit → Return*	17.0%/year	
Probability of sharing injection equipment in the previous month according to DCR attendance or not		COSINUS effectiveness results.
* DCR attendance*	9.18e-3	
* No DCR attendance*	0.111	
HIV infection rates in the absence of shared injection equipment	1.58e-3	Calibrated to obtain an initial incidence of 173/100,000 person-years [16]
Relative risk when sharing injection equipment	2.36	[17]
HCV infection rate in the absence of shared injection equipment	0.111	Calibrated to obtain an initial incidence of 11.2/100 person-years [18]
Relative risk when sharing injection equipment	1.94	[19]
Rate of abscesses according to DCR attendance or not		COSINUS results.
* DCR attendance*	0.070/year	
* No DCR attendance*	0.301/year	
Proportion of abscesses requiring hospital management	31.5%	[20]
Proportion of abscesses associated with infective endocarditis	2.2%	[21]
* With surgery*	65.8%	[22]
* Associated mortality*	5.5%	
Rate of emergency department visits according to DCR attendance or not		COSINUS results.
* DCR attendance*	0.36/year	
* No DCR attendance*	1.04/year	
Proportion of emergency department arrivals by MERS ambulance	6.3%	[23], value for Belgium
Overdose rates		
* DCR attendance*	0.018/year	
* No DCR attendance*	0.059/year	COSINUS results + 3.8% fatal overdoses [24, 25]
Proportion of overdoses leading to hospitalization	32.6%	COSINUS effectiveness results.
Proportion of fatal overdoses	3.8%	[24, 25]

*DCR* Drug consumption room, *HCV* Hepatitis C virus, *HIV* Human immunodeficiency virus

### Model description

**Fig. 1:**
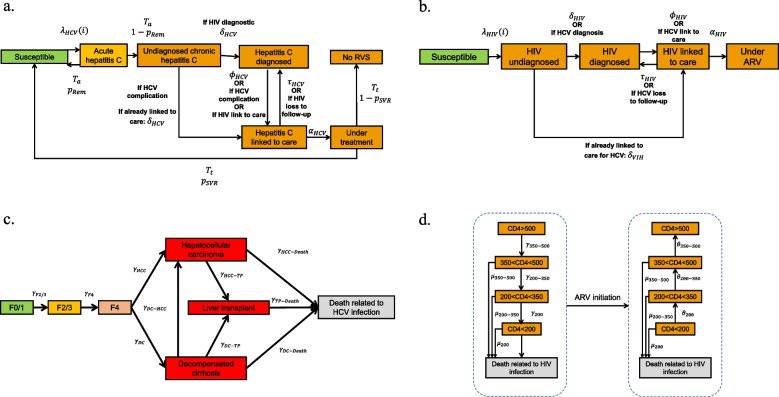
Model for a. HCV infection and chronic hepatitis C care cascade, b. HIV infection and cascade of care, c. natural history of chronic hepatitis C and d. natural history of HIV infection. ${\uplambda }_{\text{HCV}}(\text{i})$ = rate of infection according to whether or not injection equipment was shared within one month. ${\text{T}}_{\text{a}}$ = duration of acute hepatitis C. ${\text{p}}_{\text{Rem}}=$ probability of spontaneous remission. ${\updelta }_{\text{HCV}}$ = rate of HCV testing; ${\upphi }_{\text{HCV}}$ = rate of linkage to care. ${\uptau }_{\text{HCV}}$ = rate of loss to follow-up. *a*_VHC _= rate of initiation of treatment. ${\text{T}}_{\text{t}}$ = duration of antiviral therapy. ${\text{p}}_{\text{RVS}}$ = probability of sustained virological response. ${\upgamma }_{\text{F}2/3}$ rate of progression to F2/3 fibrosis. ${\upgamma }_{\text{F}4}$ = rate of occurrence of cirrhosis in F2/3. ${\upgamma }_{\text{HCC}}$ = rate of occurrence of hepatocellular carcinoma in cirrhosis. ${\upgamma }_{\text{DC }-\text{HCC}}$ = rate of occurrence of hepatocellular carcinoma in decompensated cirrhosis. ${\upgamma }_{\text{DC}}$ = rate of occurrence of decompensated cirrhosis in cirrhosis. ${\upgamma }_{\text{HCC}-\text{TP}}$ = rate of liver transplants with hepatocellular carcinoma. ${\upgamma }_{\text{DC}-\text{TP}}$ = rate of hepatic transplants with decompensated cirrhosis. ${\upgamma }_{\text{HCC}-\text{Death}}$ = death rate from hepatocellular carcinoma. ${\upgamma }_{\text{DC}-\text{Death}}$ = death rate in decompensated cirrhosis. ${\upgamma }_{\text{TP}-\text{Death}}$ = death rate after liver transplantation. ${\uplambda }_{\text{HIV}}(\text{i})$ = rate of infection by sharing or not sharing injecting equipment in the month. ${\updelta }_{\text{HIV}}$ = HIV testing rate. ${\upphi }_{\text{HIV}}$ = rate of linkage to care. ${\uptau }_{\text{HIV}}$ = rate of loss to follow-up. *a*_HIV _= rate of initiation of treatment. ${\upmu }_{\text{x}}$ = mortality rate at CD4 level x. ${\upgamma }_{\text{x}}$ = rate of decline of CD4 level to $\text{x}$. ${\uptheta }_{\text{x}}$ = rate of improvement in CD4 level towards $\text{x}$. Abbreviations: ARV=antiretrovirals; HCV=Hepatitis C virus; HIV=Human immunodeficiency virus; SVR=Sustained Virologic Response

A schematic representation of the model is given Fig. 1, and the full model structure is described in Additional file S2. The model accounted for HCV and HIV infections and simulated the cascade of care associated with both these infections (i.e., access to screening, linkage to care, treatment) and their natural history. HIV and HCV infection rates in susceptible PWID were determined by data from the COSINUS cohort on the sharing of injecting equipment depending on whether or not PWID attended a DCR. Infected PWID then progressed through the HCV and HIV cascade of care which will determine their access or not to treatment and subsequent treatment success (i.e., sustained virological response (SVR) for HCV and viral load suppression for HIV).

Furthermore, the model accounted for the occurrence of skin abscesses (which may be associated with IE) and of overdoses. Each of these events could lead to hospitalization, while IE and overdoses could lead to death. The model also included ED visits, some of which require sending a French mobile emergency and resuscitation service (MERS) ambulance.

### Outcomes

First, the following outcomes were compared between the two strategies over a 10-year period: i) morbidity, assessed using the number of HIV and HCV infections, abscesses and associated IE, overdoses and ED visits; ii) mortality, assessed using the number of deaths and life years (LY); iii) medical costs associated with each health event. We calculated health events avoided, life years saved (LYS), and associated total medical cost avoided as the difference between the number of health events/LY/medical costs for the DCR and no DCR strategies.

Second, the following three outcomes were estimated in a lifetime cost-effectiveness analysis: i) incremental costs of the DCR strategy (versus the comparator strategy) which included medical costs, initial DCR implementation costs, and DCR running costs; ii) the number of QALYs gained (preferred outcome [26]); iii) incremental cost-effectiveness ratios (ICERs) in euros per QALY. As recommended by the HAS, outcomes of the cost-effectiveness analysis were discounted at a rate of 4% per year up to 30 years, then using a linear decrease to reach 2% at 40 years [15].

### Input parameters for the model

A summary of the key parameter values and sources is provided in Table 1, and additional information is available in Additional file S3. The relative risks of abscesses, ED visits and overdoses with the DCR strategy (compared to the no DCR strategy) were estimated using data from the COSINUS cohort [11]. Using data from the literature, the proportion of abscesses associated with IE, and the proportion of ED visits associated with a MERS ambulance intervention were set at 2.2% [21] and 6.3% [23], respectively. The proportion of abscesses requiring hospital management was estimated at 31.5% [20].

The relative risk of HIV and HCV infection with the DCR strategy was estimated using information on the sharing of injecting equipment from the COSINUS cohort data, combined with values for the relative risk of HIV and HCV infection when sharing injection equipment obtained from the literature (i.e., 2.36 for HIV [17] and 1.94 for HCV [19]). HIV and HCV infection rates in the no DCR strategy were calibrated to reproduce the incidence rates observed in PWID in France prior to the opening of the DCRs in Paris and Strasbourg, i.e., 173/100,000 person-years for HIV [16], and 11.2/100 person-years for HCV [18]. All other estimates for parameters were taken from the literature.

#### Health-related quality of life

Our model accounted for the deterioration in quality of life associated with the HIV and HCV infections using utility score data from the literature based on CD4 level for HIV infection, and liver disease stage for HCV (Table 2).

**Table 2:** Effect of HIV and HCV infection on quality of life according to disease stage

**Health status**	**Marginal effect** **(QALY)**	**References**
HCV positive, fibrosis F0/F1	-0.17	[27]
HCV positive, fibrosis F2/F3	-0.18	
HCV positive, compensated cirrhosis	-0.24	
Decompensated cirrhosis/hepatocellular carcinoma	-0.40	[27, 28]
Liver transplantation		
First year	-0.45	
Subsequent years	-0.38	
After SVR at F2/F3/F4	-0.15	[28]
HIV positive, not in care, never treated with ARVs, CD>200/μL	–0.04	[29]
HIV-positive, not in care, never treated with ARVs, CD≤200/μL	–0.17	
HIV positive, linked to care, CD4>200/μL	–0.13	
HIV positive, linked to care, CD4≤200/μL	–0.18	
HIV positive, linked to care, on ARVs, CD4>200/μL	–0.11	
HIV positive, linked to care, on ARVs, CD4≤200/μL	–0.14	

In the case of HIV-HCV co-infection, we considered that the decline in quality of life due to co-infection was the sum of the individual deterioration in quality of life associated with each infection

*ARVs* Antiretrovirals, *HCV* Hepatitis C virus, *HIV* Human immunodeficiency virus, *SVR* Sustained Virological Response

#### Costs

The costs are described in Table 3 and Supplemental Material S4.

**Table 3:** Costs of Paris and Strasbourg DCRs’ implementation and medical events. More details can be found in Additional file S4

**Parameter**	**Value**	**Reference**
Costs related to the implementation of the DCR, Paris		Financial and accounting documentation of the DCRs.
* Equipment*	€44,552	
* Facilities*	€1,197638	
Costs related to the implementation of the DCR, Strasbourg		
* Equipment*	€76,558	
* Facilities*	€562,842	
Running costs of the DCR, Paris		Financial and accounting documentation for DCRs, assumption: stable from 2019.
* 2016*	€572,927	
* 2017*	€1,661,595	
* 2018*	€2,437,995	
* 2019 and after*	€2,850,265	
Running costs of the DCR, Strasbourg		
* 2017*	€1,137,743	
* 2018*	€1,165,997	
* 2019 and beyond*	€1,237,476	
Average annual costs of managing HIV infection according to disease stage		Derived from [30, 31]. The costs correspond to the costs of care for 2010.
* CD4>500*	€17,633	
* Of which treatment costs*	€13,044	
* 350<CD4<500*	€19,591	
* Of which treatment costs*	€13,514	
* 200<CD4<350*	€23,949	
* Of which treatment costs*	€14,337	
* CD4<200*	€33,834	
* Of which treatment costs*	€15,394	
Annual costs attributable to chronic hepatitis C	See Additional table A4	
Costs of antiviral therapy, chronic hepatitis C (12 weeks)	€24,900	[32]
Average cost of managing an abscess in hospital	€1,462	Average cost of care for patients group 09C101 ("Other procedures on the skin, subcutaneous tissue or breasts, level 1") and 09C10J ("Other procedures on the skin, subcutaneous tissue or breasts, outpatient") [33]
Average cost of managing infective endocarditis in hospital		[33, 34]
*Without surgery*	€12,010	
*With surgery*	€52,039	
Average cost of an emergency department visit in France	€216	[35]
Average cost of a MERS ambulance intervention	€3,154	[35, 36]
Average cost of managing an overdose in hospital	€1,483	[33, 34]

*HCV* Hepatitis C virus, *DCR* Drug Consumption Room, *MERS* Mobile Emergency and Resuscitation Service

The DCRs’ initial implementation costs and their annual running costs were estimated from the financial and accounting documents of both structures. The medical costs associated with the management of chronic HCV (according to liver disease stage) and of HIV (according to the CD4 level) were provided by the scientific and gray literature. The average costs of in-hospital management of abscesses, associated IE, overdoses and ED visits, as well as the average cost of a MERS ambulance intervention (assuming an average intervention time of one and a half hours [36]) were obtained from the French NHI hospitalization database [33–35].

All costs were inflated in 2023 euros [37].

### Economic and sensitivity analysis

#### Base-case analysis

The methods employed in the economic analysis were in line with international guidelines [38, 39]. We estimated the lifetime incremental costs and lifetime incremental health benefits of DCRs as the cost and QALY differences between the DCR and no DCR strategies. The ICER was then computed as the ratio of the incremental cost to the number of QALY gained. As the HAS does not provide recommendations on cost-effectiveness thresholds (CET) to use in France [15], we assumed the following CET suggested by the World Health Organization (WHO) in order to provide an indication on the cost-effectiveness of DCR [40]: i) very cost-effective if the ICER is less than one times the 2023 French per-capita gross domestic product (GDP) (€33,300 [41]) and ii) cost-effective if the ICER is less than three times the 2023 French per-capita GDP (€99,900). We also considered a more realistic approach to define the CET in France based on ICERs of interventions that national health authorities considered worthy of NHI funding, i.e. €50,000/QALY gained [42, 43].

In the base-case analysis, we accounted for the uncertainty related to stochasticity (i.e., the relatively small size of the simulated populations) by performing, for each strategy and for each city, 1,000 simulations for each scenario. Using the simulations, we estimated the means and associated 95% confidence intervals (CI 95%) associated with each outcome using bootstrapping (See Additional file S5 for further details).

#### Scenario and sensitivity analysis

We conducted two alternative scenario analyses. In the first, we assumed a 20% decrease in the DCRs’ entry rates after 2019 compared to the base-case analysis. In the second, we assumed that DCRs were not created as separate structures from existing harm reduction services (i.e., CAARUD) but within them.

Finally, we addressed uncertainty in the model parameters using a probabilistic sensitivity analysis (PSA) with Monte Carlo simulations including 1,000 iterations [44]. This method enables to derive the cost-effectiveness acceptability curve (See Supplemental Material S5).

### Role of the funding source

The study’s financial sponsors had no role in the design of the study. Neither were they involved in data collection, analysis or interpretation. Furthermore, they were not involved in the preparation, reviewing or approval of this manuscript.

## Results

### Base-case analysis

#### Health events, deaths and medical costs avoided over 10 years (end of 2016 to end of 2026)

The sizes of the simulated populations expected to attend the two DCRs over the 10-year period were estimated at 2,997 and 2,971 PWID in Paris and Strasbourg, respectively.

**Fig. 2:**
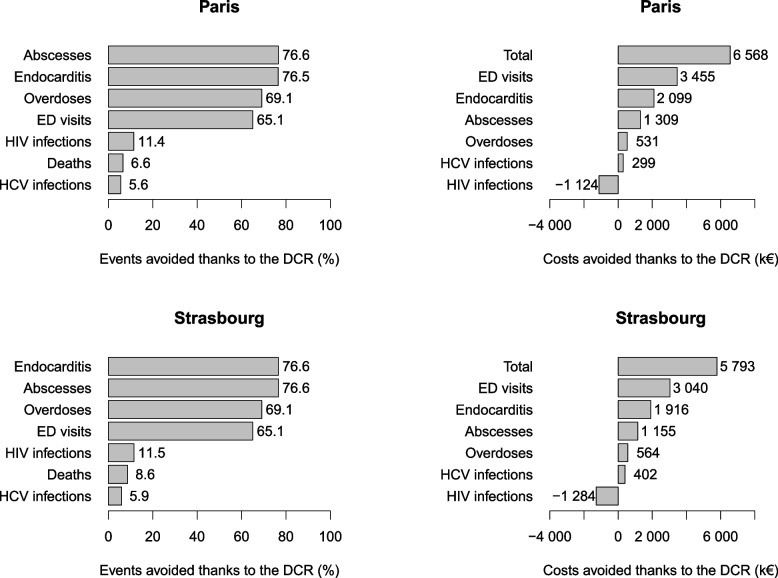
Proportion of medical events avoided (left) and medical costs avoided (right) with the DCRs in Paris (top) and Strasbourg (bottom). Abbreviations: DCR=Drug consumption room; ED=Emergency department; HCV=Hepatitis C virus; HIV=Human immunodeficiency virus

Table 4 presents the mean number [95% confidence interval – CI] of expected health events (HIV and HCV infections, abscesses and related IE, ED visits, overdoses and deaths) and mean [95% CI] expected medical costs (undiscounted) in both strategies (with/without DCR), as well as the mean number [95% CI] of health events and medical costs avoided in the DCR strategy. In addition, Fig. 2 shows the variations in the number of health events and associated costs (i.e., percentage decrease or increase) observed between both strategies.

**Table 4:** Results of main analysis for Paris and Strasbourg DCRs’ effectiveness outcomes with associated costs - means (for 1,000 simulations) and associated 95% intervals

	Paris (*n*=2,997)			Strasbourg (*n*=2,971)		
	S1 DCR	S2 No DCR	Differential S1-S2	S1 DCR	S2 No DCR	Differential S1-S2
1. Estimated number of health events in the PWID population over a 10-year DCR implementation period (end of 2016- end of 2026)						
HCV Infections	644 [642 ; 645]	682 [680 ; 683]	-38 [-40 ; -36]	565 [563 ; 566]	600 [598 ; 601]	-35 [-37 ; -33]
HIV infections	16.9 [16.7 ; 17.2]	19.1 [18.8 ; 19.3]	-2.2 [-2.5 ; -1.8]	15.1 [14.8 ; 15.3]	17 [16.8 ; 17.3]	-2 [-2.3 ; -1.6]
Abscesses	892 [891 ; 894]	3,820 [3,815 ; 3,824]	-2,927 [-2,932 ; -2,922]	797 [796 ; 799]	3,409 [3,405 ; 3,413]	-2,612 [-2,616 ; -2,607]
Emergency department visits	4,630 [4,625 ; 4,635]	13,267 [13,256 ; 13,279]	-8,637 [-8,649 ; -8,624]	4,126 [4,121 ; 4,131]	11,820 [11,811 ; 11,830]	-7,694 [-7,706 ; -7,683]
Infective endocarditis	19 [18 ; 19]	79 [78 ; 80]	-60 [-61 ; -60]	17 [16 ; 17]	71 [70 ; 71]	-54 [-55 ; -54]
Overdoses	223 [222 ; 223]	720 [718 ; 722]	-497 [-500 ; -496]	198 [197 ; 199]	641 [640 ; 643]	-443 [-445 ; -441]
Deaths	308 [307 ; 309]	330 [329 ; 331]	-22 [-23 ; -20]	196 [195 ; 196]	214 [213 ; 215]	-18 [-20 ; -17]
2. Estimated costs (lifetime) associated with each health event in the PWID population, undiscounted						
Cost - HCV (K€)	26,125 [26,051 ; 26,200]	26,424 [26,353 ; 26,495]	-299 [-391 ; -209]	28,319 [28,233 ; 28,406]	28,722 [28,630 ; 28,812]	-402 [-510 ; -295]
Cost - HIV (K€)	202,164 [201,265 ; 203,103]	201,040 [200,039 ; 202,000]	1,124 [38 ; 2,162]	232,726 [231,471 ; 233,984]	231,442 [230,239 ; 232,623]	1,284 [153 ; 2,348]
Cost - Abscesses (K€)	2,602 [2,598 ; 2,606]	3,911 [3,906 ; 3,916]	-1,309 [-1,315 ; -1,302]	3,011 [3,006 ; 3,015]	4,166 [4,161 ; 4,171]	-1,155 [-1,161 ; -1,148]
Cost – Emergency room visits (K€)	8,776 [8,764 ; 8,787]	12,230 [12,217 ; 12,244]	-3,455 [-3,473 ; -3,437]	9,980 [9,966 ; 9,992]	13,020 [13,005 ; 13,034]	-3,040 [-3,059 ; -3,022]
Cost – Infective endocarditis (K€)	4,228 [4,203 ; 4,252]	6,326 [6,299 ; 6,356]	-2,099 [-2,139 ; -2,063]	4,852 [4,823 ; 4,880]	6,767 [6,734 ; 6,799]	-1,916 [-1,959 ; -1,873]
Cost - Overdoses (K€)	241 [240 ; 242]	772 [771 ; 774]	-531 [-533 ; -530]	257 [256 ; 258]	821 [819 ; 822]	-564 [-566 ; -562]
Total medical costs (K€)	244,136 [243,197 ; 245,076]	250,704 [249,700 ; 251,672]	-6,568 [-7,690 ; -5,512]	279,144 [277,887 ; 280,406]	284,937 [283,706 ; 286,118]	-5,793 [-6,953 ; -4,674]

*DCR* Drug consumption room, *HCV* Hepatitis C virus, *HIV* Human immunodeficiency virus

The mean number of HIV infections decreased by 11.4% (in Paris) and 11.5% (in Strasbourg) in the DCR strategy (compared to the strategy without DCR) and the mean number of HCV infections by 5.6% and 5.9%, respectively. For the other health events (abscesses and related IE, overdoses, and ED visits), large reductions (i.e., between 65.1% and 76.6%) were observed in the DCR strategy compared to strategy without DCR. These avoided health events resulted in a 6.6% and 8.6% reduction in the number of deaths in Paris and Strasbourg, respectively, compared to strategy without DCR, corresponding to an increase in life expectancy of 5 and 6 months, respectively, in the DCR strategy.

The largest expected medical costs avoided concerned ED visits (Paris: k€3,455 [3,437; 3,473] / Strasbourg: k€3,040 [3,022; 3,059]), followed by IE (Paris: k€2,099 [2,063; 2,139] / Strasbourg: k€1,916 [1,873; 1,959]), and abscesses (Paris: k€1,309 [1,302; 1,315] / Strasbourg: k€1,155 [1,148; 1,161]. For HIV, the DCR strategy led to additional costs compared to the strategy without DCR (Paris: k€1,124 [38; 2,162] / Strasbourg: k1,284 [153; 2,348]). However, the total expected medical costs remained lower in the DCR strategy because of the total expected medical costs avoided, which were estimated at k€6,568 [5,512; 7,690] / k€5,793 [4,674; 6,953] in Paris and Strasbourg, respectively.

#### Cost-effectiveness of DCR over lifetime

When considering the costs of the DCRs and CAARUDs in the cost-effectiveness analysis, the total expected lifetime cost (after discounting) in the DCR strategy was higher than in the comparator strategy, corresponding to an incremental expected lifetime cost of k€16,178 [15,663; 16,700] in Paris and k€5,827 [5,291; 6,346] in Strasbourg.

In addition, in both cities, expected lifetime QALYs were also significantly higher in the DCR strategy than in the comparator, yielding 529 [492; 563] and 635 [599; 671]) QALYs gained with the DRC in Paris and Strasbourg, respectively. The ICER [95% CI] of the DCR strategy (versus the comparator strategy) was estimated at €30,600 [28,500; 33,100] and €9,200 [8,300; 10,100] per QALY in Paris and Strasbourg, respectively (See Table 5).

**Table 5:** Results of main analysis for the economic outcomes - means (for 1,000 simulations) and associated 95% intervals

		**Undiscounted**				**Discounted**			
		**Total cost** **(K€)**	**LYs** **(total)**	**QALYs** **(total)**	**Life** **expectancy**	**Total cost** **(K€, total)**	**LYs** **(total)**	**QALYs** **(total)**	**ICER** **(€/QALY)**
**Paris (*n*=2,997)**	*S1* *DCR*	270,548 [269,608 ; 271,488]	92,529 [92,469 ; 92,587]	89,084 [89,025 ; 89,141]	30.87 [30.85 ; 30.89]	150,652 [150,185 ; 151,131]	46,762 [46,738 ; 46,788]	44,847 [44,823 ; 44,872]	
	*S2* *No DCR*	250,704 [249,700 ; 251,672]	91,397 [91,332 ; 91,461]	87,927 [87,861 ; 87,991]	30.5 [30.47 ; 30.52]	134,474 [133,959 ; 134,968]	46,249 [46,222 ; 46,275]	44,318 [44,291 ; 44,344]	30,600 [28,500 ; 33,100]
**Strasbourg (*n*=2,971)**	*S1* *DCR*	291,942 [290,685 ; 293,204]	106,915 [106,848 ; 106,982]	103,063 [102,993 ; 103,130]	35.98 [35.96 ; 36.01]	149,806 [149,189 ; 150,408]	50,993 [50,966 ; 51,019]	48,973 [48,945 ; 49,000]	
	*S2* *No DCR*	284,937 [283,706 ; 286,118]	105,456 [105,390 ; 105,521]	101,581 [101,512 ; 101,649]	35.49 [35.47 ; 35.51]	143,979 [143,379 ; 144,561]	50,373 [50,346 ; 50,399]	48,338 [48,310 ; 48,364]	9,200 [8,300 ; 10,100]

*DCR* Drug consumption room, *LYs* Life years, *QALYs* Quality-adjusted life-years, *ICER* Incremental cost-effectiveness ratio

### Alternative scenarios

In the first alternative scenario (i.e., decrease in the DCRs’ new entry rates after 2019), the ICER increased to €33,900 [31,600; 36,500] per QALY in Paris and €12,000 [10,900; 13,200] per QALY in Strasbourg (after discounting), as we assumed that the implementation and running costs of DCRs would remain constant even when the population size decreased as a consequence of lower attendance rates (estimated at -20% compared to the base-case value).

In the second scenario, where we assumed that the DCRs were set up inside CAARUD, health outcomes remained unchanged but the expected incremental cost fell (after discounting) to k€11,318 [10,804; 11,839] in Paris and k€1,592 in Strasbourg [1,055; 2,111], resulting in lower discounted ICERs (i.e €21,400 [19800; 23,200] and €2,500 [1,700; 3,300] per QALY in Paris and Strasbourg, respectively).

### Sensitivity analysis

**Fig. 3:**
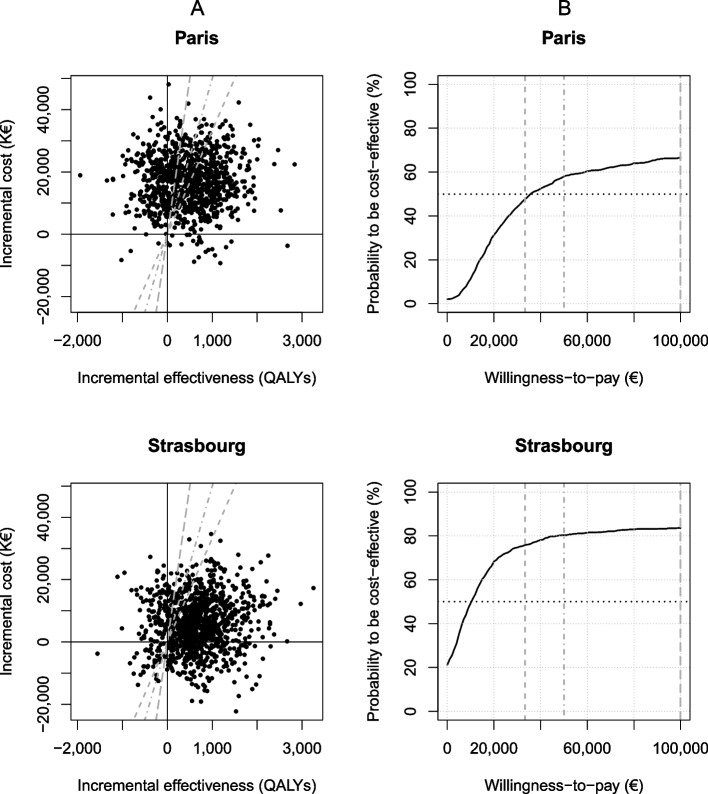
Results obtained from 1,000 Monte-Carlo simulations for the probabilistic sensitivity analysis. Each simulation is represented according to the incremental effectiveness and the incremental cost of the DCR on the cost-effectiveness plane (**A**). The acceptability curves represent the proportion of simulations below the willingness-to-pay threshold as a function of the latter (**B**). The grey short-dashed line represents one-times the French GDP per capita (€33,300); the grey long-dashed line represents three-times the French GDP per capita (€99,900). The grey dot-dashed line represents the incremental cost-effectiveness ratio of interventions adopted in France based on their cost-effectiveness (€50,900). Results are presented for Paris and Strasbourg. Abbreviations: ICER=Incremental cost-effectiveness ratio; GDP=Gross domestic product; QALY=Quality-adjusted life-year

The results of the PSA are presented in Fig. 3.

In Paris, the DCR had a 48 % probability of being very cost-effective at the WHO-recommended CET of €33,300/QALY (i.e., one times the 2023 French GDP per capita) and a 67% probability at WHO-recommended CET of €99,900/QALY (i.e. three times the 2023 French GDP per capita).

In Strasbourg, the DCR probability of being very cost-effective was 76% and the probability of cost-effective was 84% at the WHO CET of €33,300/QALY and €99,900/QALY CETs, respectively.

Using as a CET, the ICER of interventions that the national health authorities considered worthy of NHI funding (€50,000/QALY), the probability that DCR was cost-effective was 58% in Paris and 80% in Strasbourg. In addition, in Strasbourg, the DCR was cost-saving in 21% of the simulations.

## Discussion

This modelling study provides information on the projected long-term health benefits, costs and cost-effectiveness of two recently established experimental DCRs in Paris and in Strasbourg in France. Our findings highlighted that over a ten-year period, attending a DCR would significantly reduce the occurrence of health events and therefore lead to significant medical costs avoided. Interestingly, the main potential health benefits of the two DCRs in our study were fewer abscesses and associated IEs (three quarters of these events being avoided) and a reduction in ED visits and overdoses (two thirds being avoided). However, only a relatively modest number of HIV (-6%) and HCV (-11%) infections would be avoided over the ten-year period. Besides, a total of 40 deaths would be prevented over 10 years corresponding to an increase in life expectancy of 5 and 6 months in PWID attending the Paris and Strasbourg DCRs, respectively. Overall, the two DCRs would avoid €6.6 million of medical costs in Paris and €5.8 million in Strasbourg over the 10 years. These significant savings are achieved mainly through avoided ED visits and abscesses (the most frequent events), as well as avoided IE (rare but costly events) which exceed the additional costs for HIV care due to concurrent mortality avoided.

In the lifetime cost-effectiveness analysis, ICERs [95% CI] were estimated at €30,600 [28,500; 33,100] per QALY in Paris and €9,200 [8,300; 10,100] per QALY in Strasbourg in the base-case analysis, taking into account stochastic uncertainty. These findings suggest that the two DCRs would be cost-effective in both cities when considering a CET of one times the French per capita GDP (€33,300 in 2023) and when considering CETs defined based on the ICERs of interventions that the national health authorities deem to be worthy of NHI funding (i.e., €50,000/QALY).

Furthermore, the scenario analysis highlighted that the cost-effectiveness of DCRs would be significantly improved if they are established inside existing harm reduction services (specifically CAARUD) as it would considerably reduce the costs. With ICERs [95% CI] decreasing to €21,400 [19,800; 23,200] per QALY in Paris and to €2,500 [1,700; 3,300] per QALY in Strasbourg, the DCRs would be a very cost-effective intervention, particularly in Strasbourg.

Our findings are consistent with those of other cost-effectiveness studies which also demonstrated that DCRs bring important health benefits to PWID and constitute a cost-effective intervention [24, 45–57]. However, all such studies were conducted in North American settings which differed substantially from the French context [58]. First, unlike in a part North America, harm reduction services and healthcare in case of health events - including hospital care - are provided free of charge in France through the NHI. Furthermore, overdose incidence and associated mortality are much lower in France than in North America (e.g., 463 deaths from opioid overdoses reported in France in 2017 [59] *versus* 75,673 between May 2020 and April 2021 in the United States [60], a country whose population is six times greater than that of France).

Our study is the first to demonstrate the economic value of DCRs in the setting of a European country characterized by a universal health system. It also provides more comprehensive information than previous studies on the health benefits of DCR by taking into account the effects of this harm reduction intervention on the most frequent health events which PWID face, and by including all-cause ED visits, something which has not been considered to date in the literature.

Nevertheless, this study has several limitations. First, observational data on DCR attendance were only available for the period 2016-2019, and we therefore assumed that the new entry rate would remain stable after 2019. However, the sensitivity analysis highlighted that when considering a lower entry rate, the ICERs would remain acceptable at the two CET defined above. Second, as with any simulation-based analysis, there was a large degree of uncertainty over the values used for the parameters, irrespective of the sources used to define their values (i.e., scientific literature, gray literature, and the COSINUS cohort). As we used a stochastic individual-based model to assess the uncertainty related to the population size, running the model was particularly time-consuming and it was not possible to perform an extensive deterministic, univariate sensitivity analysis to identify the most sensitive parameters. However, we performed a probabilistic sensitivity analysis taking all sources of uncertainties (uncertainty over the key parameters, uncertainty caused by the small study population size, and uncertainty due to the low incidence of certain events). In that analysis, the probability of the DCR being very cost effective was close to 50 % in Paris at the CET of one time the French per capita GDP and below 80% at the CET of three times the French per capita GDP (i.e. 48% and 67%, respectively) but close to 80% in Strasbourg for both CET (76% and 84%, respectively). Furthermore, the probability of DCR being cost-effective was 58% in Paris and 80% in Strasbourg using a CET of €50,000/QALY, corresponding to the ICERs of interventions previously adopted in France by the national health authorities. These results suggest a relatively good confidence in the cost-effectiveness of the DCR in Strasbourg but some uncertainty for Paris. The uncertainty on our results could be decreased by collecting additional data to refine parameters estimates. However, this process can be costly. A value of information study could inform on the interest of such studies. Finally, our study was strongly conservative as the model did not take into account all the potential benefits of DCRs, due to a lack of available data. Attending a DCR could improve PWID quality of life, especially mental health and reduce the occurrence of other bacterial infections common in this population, such as osteomyelitis or sepsis, which may be associated with high mortality and management costs. The omission of these potential benefits may therefore have led to an underestimation of the cost-effectiveness of the intervention [61]. Furthermore, we did not take into account non-health related potential positive social effects of DCRs, for example improved access to rights and reduced delinquency, as suggested by a recent sociological study [12]. Although taking these effects into account is outside the methodological framework of medico-economic analyses, it is important to stress that these potential additional benefits increase the economic value of DCRs.

## Conclusion

Our long-term findings for experimental DRCs in France show that they are effective in terms of reductions in infections and overdoses, and are efficient at the standard cost-effectiveness threshold. The creation of a drug consumption space within pre-existing harm reduction structures would make this intervention even more cost-effective and represent a pragmatic approach to its scaling up in the future.

### Supplementary Information

Additional file 1. Additional information regarding the methodology.

## Data Availability

The datasets used and/or analysed during the current study are available from the corresponding author on reasonable request.

## Abbreviations

CI: Confidence Intervals
CAARUD: *Centre d'Accueil et d'Accompagnement à la Réduction des risques pour Usagers de Drogues* - risk reduction center for drug users in France
DCR: Drug consumption room
ED: Emergency department
GDP: Gross domestic product
IE: Infective endocarditis
ICER: Incremental cost-effectiveness ratio
HAS: *Haute Autorité de Santé *– French High Health Authority
HCV: Hepatitis C virus
HIV: Human immunodeficiency virus
LY: Life-year
LYS: Life-year saved
MERS: Mobile emergency and resuscitation service
NIH: National health insurance
OFDT: *Observatoire Français des Drogues et des Tendances addictives* - French Observatory of Drugs and Drug Addiction
PWID: People who inject drugs
QALYs: Quality-adjusted life years
SVR: Sustained virological response
