# Supplementary material for: Cost-effectiveness of drug consumption rooms in France: a modelling study
Source: BMC Public Health. 2024 May 28;24:1426. doi: 10.1186/s12889-024-18909-9 (PMC11135012; doi:10.1186/s12889-024-18909-9)
Supplement: Supplementary file 1 — Additional file 1. Additional information regarding the methodology. [file 12889_2024_18909_MOESM1_ESM.pdf]

Additional files

Additional file A1: Population

In scenario S1, the simulated population is the cohort attending the DCRs between 2016 and 2026. We start with a population of size 0 (opening of the DCRs), and introduce individuals each year according to the entry rates observed in the DCRs over the period 2016-2019: i) in Paris, 484 individuals entered between October and December 2016, 402 individuals in 2017, 235 in 2018 and 242 in 2019; ii) in Strasbourg, 153 individuals entered between November and December 2016, 257 in 2017, 227 in 2018 and 298 in 2019. In the absence of data on DCRs' attendance after 2019, we assumed in the main analysis a constant annual entry rate equal to the last observed entry rate (in 2019). Furthermore, individuals who attend the DCR at one point in time may stop attending during the simulation, and eventually become DCR users again, which makes it possible to take into account discontinuities in the use of these structures.

In the model, individuals are characterized by their age and sex (according to the data on attendance at the DCRs, see Table A1). In addition, each individual is associated with i) a health status with regard to HIV infection and the associated care cascade (Susceptible/ HIV undiagnosed/ HIV diagnosed but not linked to care/ HIV linked to care/ on ARV), and ii) a health status with respect to HCV infection and the associated care cascade (Susceptible/ Acute Hepatitis C/ Undiagnosed Chronic Hepatitis C/ Diagnosed Chronic Hepatitis C/ Hepatitis C linked to care/ On Treatment/ No SVR). These health states and the possible transitions between them are determined by the models presented below (Additional file A2).

For scenario S2, we simulated the same population, considering that 100% of the PWID do not attend the DCRs but only the classic risk reduction structures in France (i.e. CAARUDs), and therefore have levels of injection material sharing, abscesses, overdoses and emergency department visits associated with non-attendance at the DCRs (as observed in the COSINUS efficiency component).

Table A1: Distribution of individuals at model entry according to age, sex and HCV and HIV status

Parameter	Value	Reference
Age/Gender Distribution, Paris		
Men, 18 to 24 years old	0.40%	
Men, 25 to 30 years old	4.50%	
Men, 31 to 40 years old	31.40%	
Men, 41 to 50	33.90%	
Men, 50 and over	14.30%	Personal communication, Gaia.
Women, 18 to 24 years old	0.50%	
Women, 25 to 30 years old	2.10%	
Women, 31 to 40 years old	6.00%	
Women, 41 to 50	4.60%	
Women, 50 and over	2.30%	
Age/Gender Distribution, Strasbourg		
Men, 18 to 24 years old	3.3%	
Men, 25 to 30 years old	14.5%	Personal communication, Ithaca.
Men, 31 to 40 years old	33.6%	
Men, 41 to 50	22.4%	

Men, 50 and over	3.3%	
Women, 18 to 24 years old	3.3%	
Women, 25 to 30 years old	4.6%	
Women, 31 to 40 years old	9.2%	
Women, 41 to 50	5.3%	
Women, 50 and over	0.5%	
Prevalence of HIV monoinfection	1.7%	According to the literature, there is an overall HIV prevalence of 13% (1), of which 87% are HCV co-infected (2), and a chronic HCV prevalence of 43% (3).
Prevalence of HCV monoinfection	31.7%	
Prevalence of HIV/HCV co-infection	11.3%	
Initial distribution in the HIV care cascade		
Not diagnosed	3.5%	(4)
Diagnosed	5.0%	
Linked to care	11.2%	
On ARVs	80.2%	
Initial distribution in the HCV care cascade		
Acute	0%	(5)<br>By assumption for the number of acute infections: we assume that their number is negligible in the population initially.
Chronic undiagnosed	9%	
Diagnosed	11.3%	
Linked to care	16.0%	
Under treatment	2.2%	
No SVR	4.1%	
Initial distribution in the pre-diagnosis HIV natural history model		
CD4>500	31.8%	Based on data on PWID followed at the Bichat hospital in Paris and Caen, between 2010 and 2016 (personal communication).
350<CD4<500	15.9%	
200<CD4<350	23.2%	
CD4<200	29.1%	
Initial distribution in the natural history model of HIV after diagnosis		
CD4>500	56.9%	Based on data on PWID followed at the Bichat hospital in Paris and Caen, between 2010 and 2016 (personal communication).
350<CD4<500	18.8%	
200<CD4 <350	11.0%	
CD4<200	13.3%	
Initial distribution in the HCV natural history model (if infected)		
F0/F1	35%	(6)<br>By assumption for complications: we assume that their number is negligible in the initial population.
F2/F3	51%	
F4	14%	
Decompensation	0%	
Hepatocellular carcinoma	0%	
Liver transplant	0%	

Abbreviations: HCV=Hepatitis C virus; HIV=Human immunodeficiency virus; ARV=Antiretrovirals; SVR=Sustained Virological Response; PWID=People who inject drugs.

Additional file A2: Model

Level of attendance at DCRs

At the beginning of the simulation, each individual attends or does not attend a DCR depending on the scenario: for scenario S1, all individuals attend a DCR when they enter the model, with possible interruptions and resumption of attendance during the simulation: according to COSINUS data, 35% of DCRs users stop attending during the year, and 17% of users who have stopped attending return during the year. We have included these interruptions and resumption rates in scenario S1. For S2, no individual attends a DCR, and this situation remains constant over time.

HCV and HIV infection, cascades of care and natural histories of chronic hepatitis C and HIV infection

The limited time since the opening of the DCRs (2016) did not allow a direct estimate of their impact on the incidence of HCV and HIV infections, which could only be measured over the long-term. The main indicator measured in the COSINUS cohort was therefore the sharing of injection equipment in the last month, which is the main risk factor for HCV and HIV infection in this population. The modelling allows the number of infections averted to be projected, using this indicator and data from the literature on the risk of HCV and HIV infection associated with injecting equipment sharing practices.

In the COSINUS results, the probability of having shared injection equipment in the last month was estimated according to whether or not they had attended a DCR. During the simulation, for each individual and at the beginning of each month, a random draw is made to determine whether or not the individual will be sharing injection equipment in the coming month, according to the probability associated with his or her current level of attendance at the DCR. The infection rate for the coming month is then determined on the basis of whether or not the individual will be sharing injecting equipment. If there is no (reported) sharing of injecting equipment, the infection rate is set at a reference value λ_{HIV} (or λ_{HCV} depending on the infection under consideration). In the case of sharing injection equipment, a relative risk of infection by HCV and HIV is applied: according to the literature, the risk of infection is multiplied by RR_{HIV} =2.36 for HIV and RR_{HCV} =1.94 for HCV when injection equipment is shared (7,8). The values of λ_{HIV} and λ_{HCV} are calibrated to reproduce, in scenario S2, the incidence rates observed among PWID in France before the opening of DCR: I_{HIV} =173/100,000 person-years for HIV, and I_{HCV} =11.2/100 person-years for HCV (3,9), using the relationships:

$$I_{HIV} = 1 - \exp[-pRR_{HIV}\lambda_{HIV} - (1-p)\lambda_{HIV}]$$
$$I_{HCV} = 1 - \exp[-pRR_{HCV}\lambda_{HCV} - (1-p)\lambda_{HCV}]$$

Once infected, PWID will progress through the HCV and HIV care cascades. A schematic of the models is shown in Figure A1 and A3, with the different possible states and the rules for transitioning between them. The different stages of these care cascades include diagnosis of infection, linkage to care, and initiation of treatment. In the case of HCV, we added a state corresponding to acute hepatitis, which can lead to spontaneous remission in around a quarter of infections (10), as well as a compartment corresponding to the absence of a Sustained Virological Response (SVR) for PWID who have benefited from antiviral treatment but who have not been cured at the end of it. For consistency, we assumed a link between the HCV and HIV care cascades: in the case of HCV/HIV co-infections, the diagnosis of one infection automatically leads to the diagnosis of the other, as does the link to care. Similarly, the occurrence of a complication of chronic hepatitis C (hepatocellular carcinoma or decompensated cirrhosis) is assumed to automatically lead to linkage to care.

Figure A1: Model: HCV infection and the chronic hepatitis C care cascade

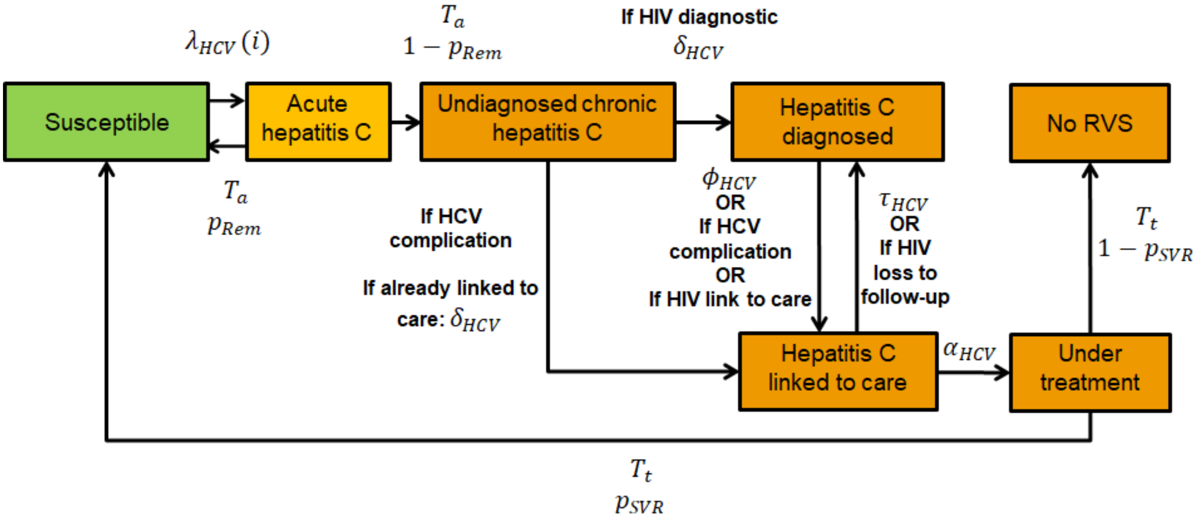

$\lambda_{HCV}(i)$ = rate of infection according to whether or not injection equipment was shared within one month. T_a = duration of acute hepatitis C. p_{Rem} = probability of spontaneous remission. δ_{HCV} = rate of HCV testing; ϕ_{HCV} = rate of linkage to care. τ_{HCV} = rate of loss to follow-up. α_{VHC}= rate of initiation of treatment. T_t= duration of antiviral therapy. p_{RVS}= probability of sustained virological response. Abbreviations: HCV=Hepatitis C virus; HIV=Human immunodeficiency virus; SVR=Sustained Virologic Response.

Figure A2: Model: Natural history of chronic hepatitis C

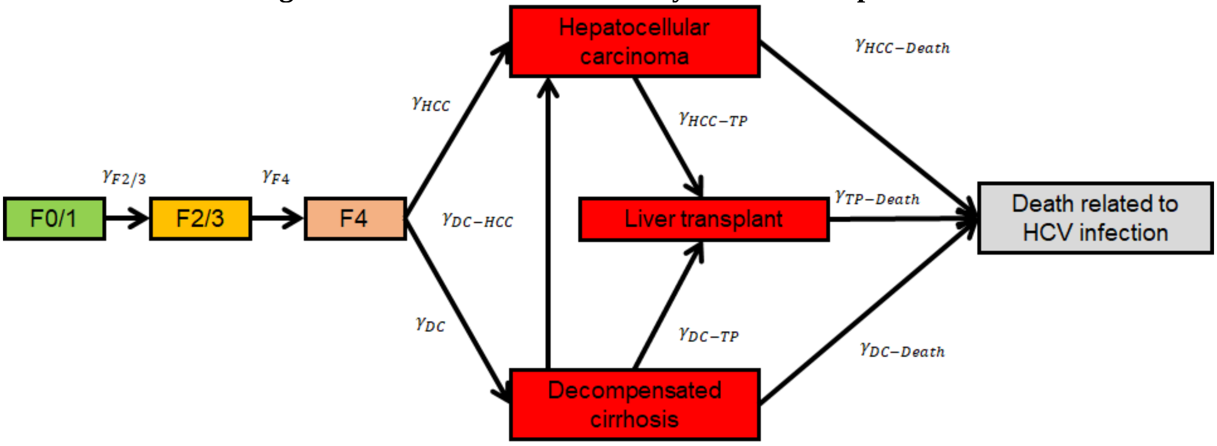

$\gamma_{F2/3}$ rate of progression to F2/3 fibrosis. γ_{F4} = rate of occurrence of cirrhosis in F2/3. γ_{HCC}= rate of occurrence of hepatocellular carcinoma in cirrhosis. γ_{DC-HCC}= rate of occurrence of hepatocellular carcinoma in decompensated cirrhosis. γ_{DC}= rate of occurrence of decompensated cirrhosis in cirrhosis. γ_{HCC-TP}= rate of liver transplants with hepatocellular carcinoma. γ_{DC-TP}= rate of hepatic transplants with decompensated cirrhosis. $\gamma_{HCC-Death}$= death rate from hepatocellular carcinoma. $\gamma_{DC-Death}$= death rate in decompensated cirrhosis. $\gamma_{TP-Death}$= death rate after liver transplantation. Abbreviations: HCV=Hepatitis C virus.

Figure A3: Model: HIV infection and HIV care cascade

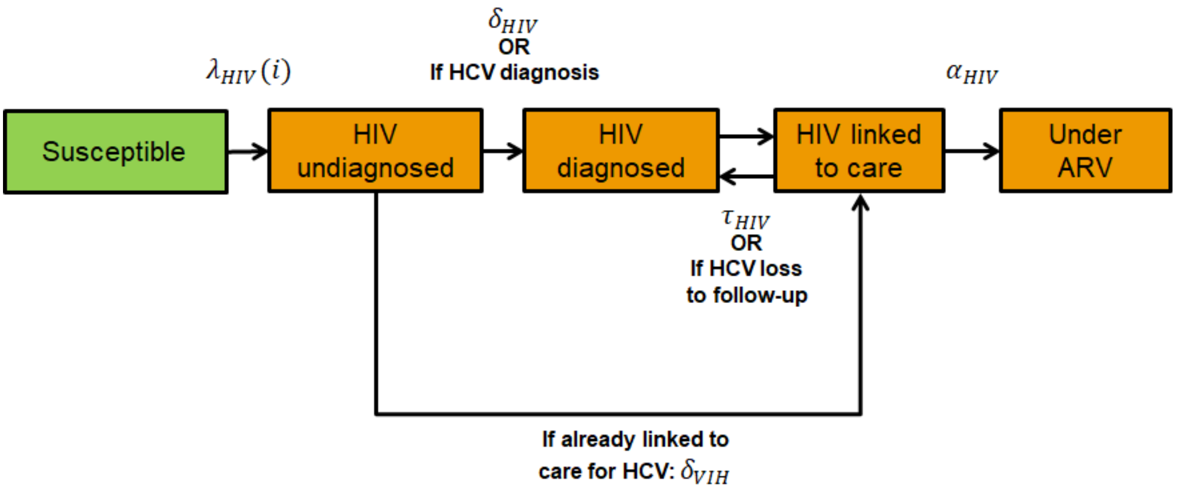

$\lambda_{HIV}(i)$ = rate of infection by sharing or not sharing injecting equipment in the month. δ_{HIV} = HIV testing rate. ϕ_{HIV} = rate of linkage to care. τ_{HIV} = rate of loss to follow-up. α_{HIV}= rate of initiation of treatment. Abbreviations: HIV=human immunodeficiency virus; ARV=antiretrovirals.

Figure A4: Model: Natural history of HIV infection

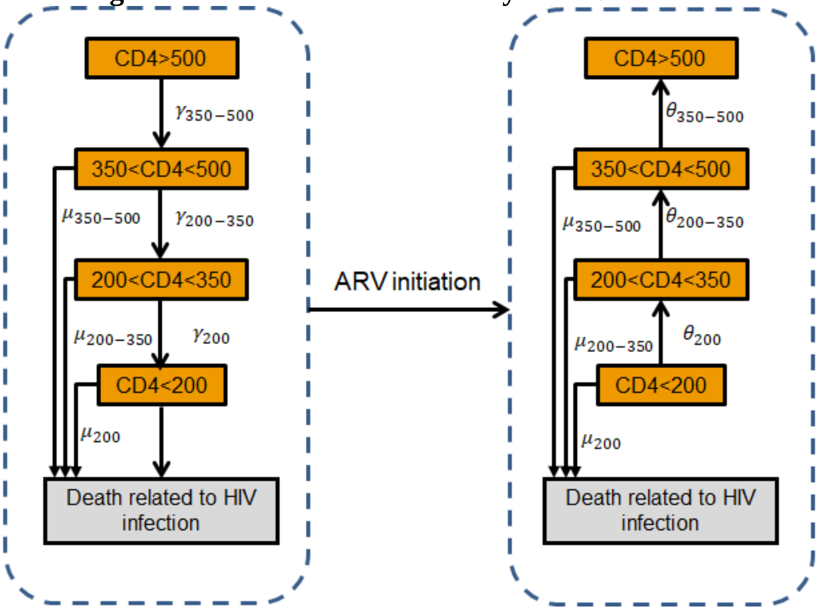

μ_x = mortality rate at CD4 level x. γ_x = rate of decline of CD4 level to x. θ_x = rate of improvement in CD4 level towards x. Abbreviations: HIV=Human Immunodeficiency Virus.

In addition, in order to simulate the mortality associated with these two infections and the costs associated with the different stages of the associated diseases, infected individuals follow an evolution in the natural history models of chronic hepatitis C and/or HIV infection (Figures A2 and A4). The course of chronic hepatitis C is described, for the early stages of the disease, by the Metavir score (from F0 to F4, the latter state corresponding to cirrhosis), and then by the potential appearance of severe complications of cirrhosis (decompensated cirrhosis or hepatocellular carcinoma), associated with high mortality. HCV progression was assumed to stop in PWID who received direct-acting antiviral treatments and achieved a SVR (i.e., HCV cured), while the disease was assumed to progress to more

advanced stages in uncured individuals. Similarly, the evolution of HIV infection is described by the patient's CD4 count (<200; 200-350; 350-500 and >500 cells), which gradually decreased in the absence of treatment, and increased again with treatment initiation (11–14). Each CD4 level is associated with a specific mortality rate.

Abscess

Abscesses occur in the population at a rate that depends on whether or not they attend a DCR and is determined from the COSINUS cohort data: the 6-month probabilities of occurrence of these events observed in COSINUS were transformed into annual rates. In addition, abscesses can be associated with complications that can be costly, notably infective endocarditis: in the case of surgical treatment in hospital, the associated costs exceeded €40,000 in 2019 (15,16). In order to take into account the impact of DCRs on these complications, a fraction of the abscesses that occur in the model are associated with infective endocarditis according to estimates obtained in the literature (17). Furthermore, each abscess or endocarditis may result in hospitalization in the model. Finally, infective endocarditis can lead to the death of the individual.

Emergency department visits

Emergency department visits are relatively inexpensive (€204 per visit on average in France (18), but frequent among PWID. Moreover, approximately 6% of PWID who present at the emergency department are brought by a Mobile Emergency and Resuscitation Service (MERS – *SMUR* in French) ambulance (19), which leads to a significant additional cost: a MERS intervention in France costed an average of €2,800 in 2019 (18,20). As for abscesses, the incidence of emergency department visits was directly measured in the efficiency component of COSINUS. The costs related to MERS interventions are also included in the model: in the absence of specific data in the COSINUS cohort, we considered that 6% of emergency department visits are associated with an MERS intervention according to the literature (19).

Overdose

The incidence of non-fatal overdoses as a function of whether or not a person attends an MRCS is assessed in COSINUS. The model also takes into account fatal overdoses, which are known to account for 3.8% of total overdoses (21,22). Each overdose can also result in a hospitalization in the model.

Substance Use and General Mortality in the PWID Population

The model also includes cessation of substance use, at a constant rate over time; and general mortality for the PWID population, dependent on the individual's infection status (active injector or not), as well as age and gender.

Additional file A3: Parameters

Parameter values for the main analysis were determined from three sources mainly:

- Data from the COSINUS cohort
- Attendance data collected by the DCRs
- Scientific literature.

Parameters are presented in Table A2, with distributions used in probabilistic sensitivity analysis.

Table A2: Model parameters

Parameter	Value	Analysis of sensitivity	Reference
Rate of new entries in the attendee population in the Paris DCR			Based on DCR attendance data available for the period 2016-2019. We assume constant entry rate from 2019 onwards in the main analysis.
2016 (October to December)	484		
2017	402		
2018	235		
2019 and beyond	242		
Rate of new entries in the attendee population in the Strasbourg DCR			
2016 (November to December)	153		
2017	257		
2018	227		
2019 and beyond	298		
Dynamics of attendance at DCRs (scenario S1)			COSINUS data, based on transitions observed between M0 and M6, and between M6 and M12.
Attendance at a DCR → Stop	35.4%/year		
Stop → Back	17.0%/year		
Probability of sharing injection equipment in the previous month according to DCR attendance or not		Triangular	COSINUS effectiveness results.
With DCR	9.18e-3	min=0 ; max=2.32e-2	
Without DCR	0.111	min=4.36e-2 ; max=0.178	
HIV infection rates in the absence of shared injection equipment	1.58e-3	Triangular - incidence min=23 ; max=315 (/105)	Calibrated to an initial incidence of 173/100,000 person-years (9).
Relative risk when sharing injection equipment	2.36	Triangular min=1.83 ; max=3.03	(8)
HCV infection rate in the absence of shared injection equipment	0.111	Triangular - Incidence: min=9/100; max=19/100	Calibrated to obtain an initial incidence of 11.2/100 person-years (3).
Relative risk when sharing injection equipment	1.94	Triangular min=1.53 ; max=2.46	(7)
Rate of abscesses according to attendance at a DCR		Triangular	COSINUS efficiency results.
DCR attendance	0.070/year	min=0.017 ; max=0.124	
No DCR attendance	0.301/year	min=0.189 ; max=0.421	

Proportion of abscesses requiring hospital treatment	31.5%	Beta(51, 109)	(23)
Proportion of abscesses associated with infective endocarditis	2.2%	Triangular<br>min=0.015 ; max=0.03	(17)
Of which requiring surgery	65.8%	Beta(43, 30)	(24)
Associated mortality	5.5%	Beta(4, 69)	
Rate of emergency department visits according to DCR attendance or not		Triangular	COSINUS results.
DCR attendance	0.36/year	min=0.095 ; max=0.557	
No DCR attendance	1.04/year	min=0.79 ; max=1.476	
Proportion of emergency department arrivals by MERS ambulance	6.3%	Beta (6, 89)	(19), value for Belgium
Overdose rates		Triangular - overdose rate non-fatal	COSINUS results + 3.8% fatal overdoses (21,22) .
DCR attendance	0.018/year	min=0 ; max=0.039 ; mode=0.017	
No DCR attendance	0.059/year	min=0.012 ; max=0.104 ; mode=0.057	
Proportion of overdoses leading to hospitalization	32.6%	Beta(33, 68)	COSINUS efficiency results.
Proportion of fatal overdoses	3.8%	Triangular<br>min=0.031 ; max=0.069	(21,22)
Duration of acute hepatitis C	0.5 years		(10)
Probability of spontaneous remission in acute hepatitis C	26%		(10)
Mean time from HCV infection to diagnosis	1.25 years		(5)
Average time from HCV diagnosis to linkage to care	2.6 years		(5)
Loss of sight rate for HCV (if linked to care)	3.4%/year		Based on data on PWID followed at the Bichat hospital in Paris and Caen, between 2010 and 2016 (personal communication).
Median time from linkage to care to initiation of antiviral therapy for HCV	0.3 years		Hypothesis, based on time to initiation of ARV treatment for HIV (4).
Duration of HCV treatment	12 weeks		(25)
Probability of SVR	95%		(25)
Transition rate, natural history model of chronic hepatitis C			
F0/F1→F2/F3	0.049/year if monoinfected<br>0.059/year if HIV co-infected		(26)

F2/F3→F4	0.051/year if monoinfected<br>0.060/year if HIV co-infected	
F4→Decompensation	0.04/year before SVR<br>5e-4 after SVR	(27,28)
F4→HCC	0.021/year before SVR<br>5e-3 after SVR	
Decompensation→HCC	0.021/year	Assumption: same as F4, based on (27,28)
Decompensation→Death	0.306/year	(27,28)
HCC→Death	0.344/year	
Decompensation →Transplantation	0.128/year	(29,30)
HCC→Transplantation	0.186/year	
Transplantation→Death	0.0174 the first year<br>0.128/year in subsequent years	
Median time from HIV infection to diagnosis	4 years	(4)
Median time from HIV diagnosis to linkage to care	0.9 years	(4)
Loss of follow-up rate for HIV (if related to care)	3.4%/year	Based on data on PWID followed at the Bichat hospital in Paris and Caen, between 2010 and 2016 (personal communication).
Median time from linkage to care to initiation of antiviral therapy for HIV	0.3 years	(4)
Transition rate, natural history model of HIV infection without ARVs		
CD4>500 → 350<CD4<500	1.01/year	(11), based on the most representative age/sex characteristics of our population.
350<CD4<500 → 200<CD4<350	0.271/year	
CD4>200 → CD4<200	0.142/year	
Transition rate, natural history model of HIV infection on ARVs		
350<CD4<500 → CD4>500	0.550/year	(31)
200<CD4<350 → 350<CD4<500	0.408/year	
CD4<200 → CD4>200	0.479/year	
Death rate from HIV infection, excluding ARVs		

350<CD4<500 → Death	0.022/year	(32)
200<CD4<350 → Death	0.033/year	
CD4<200 → Death	0.150/year	
Rate of death from HIV infection on ARVs		
350<CD4<500 → Death	0.012/year	(32)
200<CD4<350 → Death	0.018/year	
CD4<200 → Death	0.083/year	
Average time to drug cessation	13.9 years	(33)
Mortality rate		
Injector/injector inactive	Assumption: general population mortality, see Table A3	
Relative risk, active injector	5.19	(34)
Relative risk, active injector	9.52	

Abbreviations: ARV=Antiretrovirals; DCR=Drug Consumption Room; HCC=Hepatocellular carcinoma; HCV=Hepatitis C virus; HIV=Human immunodeficiency virus; MERS= Mobile Emergency and Resuscitation Service; SVR=Sustained Virological Response.

Table A3: Mortality according to age and sex in the French general population (/1,000 inhabitants) (35).

	Men	Women
**18 to 19**	0.3	0.1
**20 to 24**	0.6	0.2
**25 to 29**	0.7	0.2
**30 to 34**	0.8	0.3
**35 to 39**	1.1	0.5
**40 to 44**	1.7	0.8
**45 to 49**	2.7	1.4
**50 to 54**	4.4	2.3
**55 to 59**	7.0	3.5
**60 to 64**	10.9	5.0
**65 to 69**	14.9	6.8
**70 to 79**	25.2	13.2
**80 to 89**	75.3	49.8
**90 to 110**	220.0	181.0

Additional file A4: Costs

The costs of setting up the DCRs and their annual running costs were estimated from the financial and accounting documents of these structures. These included capital costs (building renovation or construction, furniture, and medical and IT equipment) and recurrent costs (personnel, communication, energy, injection equipment, HIV/HCV tests and supplies). The yearly economic value of capital assets was computed based on their acquisition date and expected shelf life. We considered a 7-year annuitization period for the equipment and a 15-year depreciation period for the facilities.

The medical costs associated with the management of chronic hepatitis C (according to the stage of the disease), HIV (according to the CD4 level) and the average cost of a visit to an emergency department were obtained from the scientific and grey literature. The average costs of in-hospital management of abscesses, infective endocarditis and overdoses, as well as the average cost of an emergency department visit, were obtained from the Programme du Médicalisation des Systèmes d'Information (PMSI). The average cost of an MERS intervention was extrapolated from the average pricing of the half-hour intervention, based on interventions lasting an average of one hour and a half (20).

The costs are detailed in Tables A4 and A5, with distributions used in probabilistic sensitivity analysis. All cost were inflated in 2023 euros (36).

Table A4: Costs used in the simulation.
Costs were inflated in 2023 euros.

Parameter	Value	Analysis of sensitivity	Reference
Costs related to the implementation of the DCR, Paris			
Equipment	€44,552		
Facilities	€1,197,638		Financial and accounting documentation of the DCRs.
Costs related to the implementation of the DCR, Strasbourg			
Equipment	€76,558		
Facilities	€562,842		
Running costs of the DCR, Paris			
2016	€572,927		
2017	€1,661,595		
2018	€2,437,995		
2019 and beyond	€2,850,265		Financial and accounting documentation for DCRs, assumption: stable from 2019.
Running costs of the DCR, Strasbourg			
2017	€1,137,743		
2018	€1,165,997		
2019 and beyond	€1,237,476		
Average annual costs of managing HIV infection according to disease stage			Derived from (37,38). The costs correspond to the costs of care for 2010.
CD4>500			
Of which treatment costs			
350<CD4<500	€17,633		

Of which treatment costs	€13,044		
200<CD4<350	€19,591		
Of which treatment costs	€13,514		
CD4<200	€23,949		
Of which treatment costs	€14,337		
	€33,834		
	€15,394		
Costs of antiviral therapy, chronic hepatitis C (12 weeks)	€24,900		(39)
Average cost of managing an abscess in hospital	€1,462	Triangular<br>min=1,244, max=1,988	Average cost of care for patients group 09C101 ("Other procedures on the skin, subcutaneous tissue or breasts, level 1") and 09C10J ("Other procedures on the skin, subcutaneous tissue or breasts, outpatient") (15).
Average cost of managing infective endocarditis in hospital		Triangular	(15,16)
Without surgery	€12,010	min=11,020; max=13,607	
With surgery	€52,039	min=46,120; max=59,101	
Average cost of an emergency department visit in France	€216	Triangular<br>min=170 ; max=294	(18)
Average cost of a MERS ambulance intervention	€3,154	Triangular<br>min=2,366 ; max=3 ,377	(18,20)
Average cost of managing an overdose in hospital	€1,483	Triangular<br>min=1,053; max=2,285	(15,16)
Average running cost of CAARUD (per user)	€467		Observatoire français des frogues et des tendances addictives, personnal communication

Abbreviations: CAARUD=Harm reduction centers in France; DCR=Drug consumption room; HCV=Hepatitis C virus; HIV=Human immunodeficiency virus; MERS= Mobile Emergency and Resuscitation Service.

Table A5: Annual costs attributable to chronic hepatitis C: outpatient costs (before treatment or after treatment failure) and hospital costs (no death or death in hospital) (40). Costs were inflated in 2023 euros.

Stage of liver fibrosis	Outpatient costs (euros)		Hospital costs (euros)	
	Never treated	After treatment failure	No death	Death in hospital
F0/F1	85	64	337	409
F2/F3	155	104		
F4	276	86	1,572	7,834
Decompensated cirrhosis	116		11,993	19,579
Hepatocellular carcinoma	116		14,266	20,215
Liver transplantation				
First year	116		68,046	110,184
Subsequent years	116		6,613	19,326

Additional file A5: Analysis

Base-case analysis

In the base-case analysis, we accounted for the uncertainty related to stochasticity (i.e., the relatively small size of the simulated populations) by performing, for each strategy and for each city, 1000 simulations for each scenario. More precisely, for each individual, we randomly selected individual characteristics at model entry and the dynamic of DCR attendance over the 10-year period, both of which determine the probability of sharing injecting equipment and the risk of infection, and for each time t over the 10 year-period we simulated HCV and HIV infections, abscesses and associated IE, ED visits, overdoses. Each simulation allowed us to obtain the set of outcomes of interest including: i) the number of deaths, health events and related costs (estimated over a 10-year period after the DRC opening), and ii) QALYs, costs and ICER (estimated over the cohort lifetime in the economic analysis). Using the simulations, we estimated the means and associated 95% confidence intervals (CI 95%) associated with each outcome using bootstrapping (1,000 replications).

Probabilistic sensitivity analysis (PSA)

We addressed uncertainty in the model parameters using a probabilistic sensitivity analysis (PSA) with Monte Carlo simulations including 1,000 iterations (41). We randomly drew key parameters values from their predefined distribution (described in Tables A2 and A5) and then ran simulations using the selected parameter sets for each strategy (i.e., DCR versus no DCR). These randomly drawn parameter values therefore yielded 1,000 estimates of costs and effectiveness (QALYs) which were then used to calculate the probability of each DCR being cost-effective for various CET. This probability was simply the proportion of incremental cost-effect pairs that fell below the CET represented in the cost-effectiveness plan by the line drawn through the origin. We derived the corresponding cost-effectiveness acceptability curve by plotting this probability on the y-axis versus the CET on the x-axis.

References

1. Weill-Barillet L, Pillonel J, Semaille C, Léon L, Le Strat Y, Pascal X, et al. Hepatitis C virus and HIV seroprevalences, sociodemographic characteristics, behaviors and access to syringes among drug users, a comparison of geographical areas in France, ANRS-Coquelicot 2011 survey. Rev Epidemiol Sante Publique. 2016 Sep;64(4):301–12.

2. Roux P, MARCELLIN F, LERT F, SPIRE B, DRAY SPIRA R. La co-infection par le virus de l'hépatite C chez les personnes infectées par le VIH: données de l'enquête ANRS-Vespa2. Bull Épidémiologique Hebd. 2013;(26–27):314–20.

3. Leon L, Kasereka S, Barin F, Larsen C, Weill-Barillet L, Pascal X, et al. Age- and time-dependent prevalence and incidence of hepatitis C virus infection in drug users in France, 2004-2011: model-based estimation from two national cross-sectional serosurveys. Epidemiol Infect. 2017;145(5):895–907.

4. Supervie V, Marty L, Lacombe JM, Dray-Spira R, Costagliola D, FHDH-ANRS CO4 study group. Looking Beyond the Cascade of HIV Care to End the AIDS Epidemic: Estimation of the Time Interval From HIV Infection to Viral Suppression. J Acquir Immune Defic Syndr 1999. 2016 01;73(3):348–55.

5. Cousien A, Tran VC, Deuffic-Burban S, Jauffret-Roustide M, Dhersin JS, Yazdanpanah Y. Hepatitis C treatment as prevention of viral transmission and liver-related morbidity in persons who inject drugs. Hepatol Baltim Md. 2016 Apr;63(4):1090–101.

6. Melin P, Chousterman M, Fontanges T, Ouzan D, Rotily M, Lang JP, et al. Effectiveness of chronic hepatitis C treatment in drug users in routine clinical practice: results of a prospective cohort study. Eur J Gastroenterol Hepatol. 2010 Sep;22(9):1050–7.

7. Pouget ER, Hagan H, Des Jarlais DC. Meta-analysis of hepatitis C seroconversion in relation to shared syringes and drug preparation equipment. Addict Abingdon Engl. 2012 Jun;107(6):1057–65.

8. Roy E, Richer I, Morissette C, Leclerc P, Parent R, Claessens C, et al. Temporal changes in risk factors associated with HIV seroconversion among injection drug users in eastern central Canada. AIDS Lond Engl. 2011 Sep 24;25(15):1897–903.

9. Ndawinz JDA, Costagliola D, Supervie V. New method for estimating HIV incidence and time from infection to diagnosis using HIV surveillance data: results for France. AIDS. 2011 Sep;25(15):1905–13.

10. Micallef JM, Kaldor JM, Dore GJ. Spontaneous viral clearance following acute hepatitis C infection: a systematic review of longitudinal studies. J Viral Hepat. 2006 Jan;13(1):34–41.

11. Lodi S, Phillips A, Touloumi G, Geskus R, Meyer L, Thiébaut R, et al. Time from human immunodeficiency virus seroconversion to reaching CD4+ cell count thresholds <200, <350, and <500 Cells/mm^3: assessment of need following changes in treatment guidelines. Clin Infect Dis Off Publ Infect Dis Soc Am. 2011 Oct;53(8):817–25.

12. Mocroft A, Phillips AN, Gatell J, Ledergerber B, Fisher M, Clumeck N, et al. Normalisation of CD4 counts in patients with HIV-1 infection and maximum virological suppression who are taking combination antiretroviral therapy: an observational cohort study. The Lancet. 2007 Aug 4;370(9585):407–13.

13. Wolbers M, Babiker A, Sabin C, Young J, Dorrucci M, Chêne G, et al. Pretreatment CD4 cell slope and progression to AIDS or death in HIV-infected patients initiating antiretroviral therapy--the

CASCADE collaboration: a collaboration of 23 cohort studies. PLoS Med. 2010 Feb 23;7(2):e1000239.

14. SMART Study Group, El-Sadr WM, Grund B, Neuhaus J, Babiker A, Cohen CJ, et al. Risk for opportunistic disease and death after reinitiating continuous antiretroviral therapy in patients with HIV previously receiving episodic therapy: a randomized trial. Ann Intern Med. 2008 Sep 2;149(5):289–99.

15. Etudes nationales de coûts sanitaires [Internet]. [cited 2020 Aug 17]. Available from: https://www.scansante.fr/applications/enc-mco

16. MCO par diagnostic ou acte | Stats ATIH [Internet]. [cited 2021 Feb 22]. Available from: https://www.scansante.fr/applications/statistiques-activite-MCO-par-diagnostique-et-actes

17. Spijkerman IJ, van Ameijden EJ, Mientjes GH, Coutinho RA, van den Hoek A. Human immunodeficiency virus infection and other risk factors for skin abscesses and endocarditis among injection drug users. J Clin Epidemiol. 1996 Oct;49(10):1149–54.

18. Référentiel de coût des unités d'oeuvres (RTC) | Stats ATIH [Internet]. [cited 2024 Mar 5]. Available from: https://www.scansante.fr/applications/cout-dunites-doeuvre/

19. Denis B, Dedobbeleer M, Benabderrazik A, Bizimungu DG, Sciera V. Les usagers de drogues suivis en médecine générale: recours fréquent aux services d'urgence mais pas toujours les bienvenus. Santé. 2009;(47).

20. Borthomieu L. Evaluation de l'activité des Médecins Correspondants SAMU et retour d'expérience des acteurs intervenant dans ce dispositif en Vienne entre 2015 et 2018. [PhD Thesis]. Université de Poitiers; 2018.

21. Hood JE, Behrends CN, Irwin A, Schackman BR, Chan D, Hartfield K, et al. The projected costs and benefits of a supervised injection facility in Seattle, WA, USA. Int J Drug Policy. 2019;67:9–18.

22. Neale J. A response to Darke et al., 'The ratio of non-fatal to fatal heroin overdose'. Addict Abingdon Engl. 2003 Aug;98(8):1171.

23. Hope VD, McVeigh J, Marongiu A, Evans-Brown M, Smith J, Kimergård A, et al. Injection site infections and injuries in men who inject image- and performance-enhancing drugs: prevalence, risks factors, and healthcare seeking. Epidemiol Infect. 2015 Jan;143(1):132–40.

24. Foisel C. Endocardite infectieuse du sujet toxicomane [PhD Thesis]. UHP-Université Henri Poincaré; 2009.

25. Kish T, Aziz A, Sorio M. Hepatitis C in a New Era: A Review of Current Therapies. Pharm Ther. 2017 May;42(5):316.

26. Thein HH, Yi Q, Dore GJ, Krahn MD. Estimation of stage-specific fibrosis progression rates in chronic hepatitis C virus infection: a meta-analysis and meta-regression. Hepatol Baltim Md. 2008 Aug;48(2):418–31.

27. Salomon JA, Weinstein MC, Hammitt JK, Goldie SJ. Empirically Calibrated Model of Hepatitis C Virus Infection in the United States. Am J Epidemiol. 2002 Oct 15;156(8):761–73.

28. Salomon JA, Weinstein MC, Hammitt JK, Goldie SJ. Cost-effectiveness of Treatment for Chronic Hepatitis C Infection in an Evolving Patient Population. JAMA. 2003 Jul 9;290(2):228–37.

29. Deuffic-Burban S, Mathurin P, Pol S, Larsen C, Roudot-Thoraval F, Desenclos JC, et al. Impact of hepatitis C triple therapy availability upon the number of patients to be treated and associated costs in France: a model-based analysis. Gut. 2012 Feb;61(2):290–6.

30. Mourad A, Deuffic-Burban S, Ganne-Carrié N, Renaut-Vantroys T, Rosa I, Bouvier AM, et al. Hepatocellular carcinoma screening in patients with compensated hepatitis C virus (HCV)-related cirrhosis aware of their HCV status improves survival: a modeling approach. Hepatol Baltim Md. 2014 Apr;59(4):1471–81.

31. Sucharitakul K, Boily MC, Dimitrov D, Mitchell KM. Influence of model assumptions about HIV disease progression after initiating or stopping treatment on estimates of infections and deaths averted by scaling up antiretroviral therapy. PloS One. 2018;13(3):e0194220.

32. Lewden C, Chene G, Morlat P, Raffi F, Dupon M, Dellamonica P, et al. HIV-infected adults with a CD4 cell count greater than 500 cells/mm3 on long-term combination antiretroviral therapy reach same mortality rates as the general population. J Acquir Immune Defic Syndr 1999. 2007 Sep 1;46(1):72–7.

33. Fazito E, Cuchi P, Mahy M, Brown T. Analysis of duration of risk behaviour for key populations: a literature review. Sex Transm Infect. 2012 Dec;88 Suppl 2:i24-32.

34. Lopez D, Martineau H, Palle C. Mortalité liée aux drogues illicites. Étude d'une cohorte rétrospective de personnes interpellées pour usage de stupéfiants. Saint-Denis La Plaine: Observatoire français des drogues et des toxicomanies; 2004. 158 p.

35. Ined - Institut national d'études démographiques [Internet]. [cited 2020 Aug 18]. Taux de mortalité par sexe et âge. Available from: https://www.ined.fr/fr/tout-savoir-population/chiffres/france/mortalite-cause-deces/taux-mortalite-sexe-age/

36. Institut national de la statistique et des études économiques. Convertisseur franc-euro [Internet]. [cited 2024 Mar 6]. Available from: https://www.insee.fr/fr/information/2417794

37. Sloan CE, Champenois K, Choisy P, Losina E, Walensky RP, Schackmanj BR, et al. Newer drugs and earlier treatment: Impact on lifetime cost of care for HIV-infected adults. AIDS Lond Engl. 2012 Jan 2;26(1):45–56.

38. Papot E, Landman R, Louni F, Charpentier C, Peytavin G, Certain A, et al. Budget impact of antiretroviral therapy in a French clinic cohort. AIDS Lond Engl. 2017 Jun 1;31(9):1271–9.

39. Assurance Maladie. Base de données des médicaments et informations tarifaires [Internet]. [cited 2024 Mar 6]. Available from: http://www.codage.ext.cnamts.fr/codif/bdm_it/

40. Schwarzinger M, Deuffic-Burban S, Mallet V, Pol S, Pageaux GP, Canva-Delcambre V, et al. Lifetime costs attributable to chronic hepatitis C from the French healthcare perspective (ANRS n° 12188). J Hepatol. 2013;58:S21–2.

41. Briggs AH, Weinstein MC, Fenwick EAL, Karnon J, Sculpher MJ, Paltiel AD, et al. Model parameter estimation and uncertainty analysis: a report of the ISPOR-SMDM Modeling Good Research Practices Task Force Working Group-6. Med Decis Mak Int J Soc Med Decis Mak. 2012 Oct;32(5):722–32.
